# Early age at menarche and its associated factors in school girls (age, 10 to 12 years) in Bangladesh: a cross-section survey in Rajshahi District, Bangladesh

**DOI:** 10.1186/s40101-020-00218-w

**Published:** 2020-03-23

**Authors:** Jannatul Maowa Malitha, Md. Ariful Islam, Saima Islam, Abu Sayed Md. Al Mamun, Suman Chakrabarty, Md. Golam Hossain

**Affiliations:** 1 Department of Statistics, University of Rajshahi, Rajshahi, 6205 Bangladesh; 2 Australian Research Centre for Population Oral Health (ARCPOH), The University of Adelaide, Adelaide, South Australia 5005 Australia; 3 Department of Anthropology, Mrinalini Datta Mahavidyapith, Vidyapith Road, Birati, Kolkata, 700 051 India

**Keywords:** Age at menarche, School girls, Rajshahi District, Independent sample *t* test, Logistic regression model

## Abstract

**Background:**

Early onset of menarche is one of the most important factors for breast cancer and other associated health hazards. The aim of this study was to investigate the early age at menarche and its associated factors in school girls (age, 10–12 years) in Rajshahi District, Bangladesh.

**Methods:**

Data was collected from Rajshahi District, Bangladesh, using multistage random sampling. Independent sample *t* test and binary logistic regression model were used in this study. A total number of 386 school girls aged 10–12 years were considered as a sample for this study.

**Results:**

This study revealed that more than 48% girls already attained menarche within the age of 12 years, among them 25.6%, 41.0%, and 58.3% girls experienced menarche at the age of 10, 11, and 12 years, respectively. It was observed that the menarcheal girls were significantly taller (*p* < 0.01) and heavier (*p* < 0.01) than non-menarcheal girls. The menarcheal girls’ mothers were heavier (*p* < 0.01), shorter (*p* < 0.01), had more BMI (*p* < 0.01), reached menarche (*p* < 0.05) earlier than non-menarcheal girls’ mothers. Menarcheal girls had less number of siblings (*p* < 0.01) and lower order of birth (*p* < 0.05) than non-menarcheal girls. After controlling the effect of other factors, multiple logistic regression model demonstrated that obese girls were more likely to attain menarche than under- [AOR = 0.279, CI 95% 0.075–0.986; *p* < 0.05] and normal [AOR = 0.248, CI 95% 0.082–0.755; *p* < 0.05] weight girls. Urban school girls had more chance to get menarche than rural school girls at same age (AOR = 0.012, 95% CI 0.003–0.047; *p* < 0.01).

**Conclusions:**

Therefore, modern lifestyle changes may have the important factors for early age at menarche of the studied girls in Bangladesh.

## Background

The first menstrual bleeding is referred to as menarche, and age at menarche is the most significant period of a girl’s life, and it is a part of complex process of physical and emotional development of a girl. Menarche is the most commonly remembered milestone of puberty for most women, and it is often considered the signals of fertility from both social and medical perspectives. Girls experience menarche at different ages but usually it occurs between the ages of 10 and 16 years, and the mean age at menarche varies significantly by geographical region, race, ethnicity, and other characteristic factors, especially nutritional factors [1, 2]. Recently, it was found that estradiol is an important factor for the occurrence of kisspeptin expression in GnRH (gonadotropin releasing hormone) neurons in the prepubertal period, and its gradual development provides a GnRH neuron amplification mechanism that is used to facilitate the emergence of pulsatile gonadotropin secretion necessary for puberty onset. It was also found that a single nucleotide polymorphism of *LIN28B* on chromosome 6 was associated with earlier menarche [3]. Besides genetics, menarche is also influenced by hereditary [4], early biological maturation, and many socioeconomic and environmental factors [5]. It has been reported that early onset of menarche is one of the important indicators for getting some diseases such as breast cancer [6] and ischemic heart disease [7]. Recently, Won et al. found that early menarche was significantly associated with hypertension, diabetes, and metabolic syndrome of Korean women [8]. Fida et al. reported that women who had early menarche (< 12 years old) were more likely to get asthma than their counterparts who got menarche (≥ 12 years old) [9]. It has been also found that delayed menarche is associated with some complications such as irregular menstrual cycles, low peak bone mass [10], and type I diabetes [11]. The nature of the relationship between menarcheal age and anthropometric measures may be important in understanding the significance of the effects of menarcheal age on disease in later life of women. Numerous researchers have shown that age at menarche is associated with height, weight, body mass index, and socio-economic and demographic factors [12, 13]. There is a huge number of studies available with menarcheal age of adolescent girls worldwide. With respect to Bangladeshi populations, researchers have studied menstruation among Bangladeshi females, and they have tried to find the relationship between age at menarche and nutritional status, post-menarcheal growth, marriage, anthropometric measures, and socio-demographic factors [14, 15]. Most of the studies in Bangladesh have been done with adult women. The educational attainment of a population is an important indicator of the society’s stock of human capital and level of socioeconomic development. The literacy rate especially women education level and family wealth quintile have been increasing during the last two decades in Bangladesh [16]. The mean age of menarche has declined over the last century all over the world especially in developed and developing countries. It is important to study on puberty status (age at menarche) among school going girls (age 10–12 years), and determine the risk factors for early age at menarche, in order to ensure corrective measures can be undertaken. Some studies with other population already have found that recently some girls got menarche at age 10 years [1, 17]. In this study, we considered school girls with age of 10–12 years for investigating their menstrual characteristics and to determine factors of early onset of menarche.

The aim of the study was to investigate the early age at menarche and its associated factors among school going girls aged 10–12 years in Rajshahi District, Bangladesh.

## Materials and methods

### Materials

The target area for this study was Rajshahi District, which is situated in the north-western part of the Bangladesh. The total area of the district is 2425.37 km^2^ and the population is 2,699,688 according to BBS (2011, adjusted on 16 March 2012) including the urban and rural population of the district. The population of this study was school girls with age at 10 to 12 years in Rajshahi District, Bangladesh. The subjects were all of Bangladeshi birth. The present study was a cross-sectional study. The data was collected from the field survey. It was conducted from March to July 2017.

### Methods

#### Sample size determination

The following formula was used to calculate the required sample size for this study:

$n=\frac{z^2p\left(1-p\right)}{d^2}$, where *n* = the number of samples, *z* = 1.96 for 95% confidence interval, *p* = 0.5, and finally 5% margin of error (*d* = 0.05) were considered. The above formula provided that 385 was the required sample size for the present study. However, initially, we considered 400 (4% extra) samples for assuming non-response rate. Unfortunately, 14 selected girls were not interested to provide their information. Finally, 386 samples were considered in this study and their information was collected by the principal author.

#### Sample selection procedures

Multistage random sampling technique was utilized for selecting sample covering all the population from three upazilas (the third largest administrative division in Bangladesh) of Rajshahi district. There were 9 upazilas of Rajshahi District. In the first stage, 3 upazilas were selected from 9 upazilas by simple random sampling. In the second stage, 3 schools were selected from each upazila by random sampling. Total selected schools were 3 × 3 = 9. In the third stage, 400 girls were selected by random sampling with proportion allocation technique. Principal author described the objective of the present study with selected girls. Written consent of each selected girl and school authorities’ permission had been taken. A standard questionnaire was distributed to 386 girls for getting their socio-economic and demographic information.

#### Outcome variable

The outcome variable of this study was puberty status (age at menarche) of school girls aged 10–12 years. Initially girls were asked, “Did you have experience menstruation?” If yes, then asked, when (age in year) did you attain menarche? The information was double checked over phone from the girl’s mother. The sample was classified into two classes according to their menstrual status such as (i) menarcheal girls (code, 1), (ii) non-menarcheal girls (code, 0).

#### Independent variables

Parents’ socio-economic and demographic factors and girls’ anthropometric measures were considered as independent variables in this study. The categorical variables were type of residence (rural and urban), mode of delivery (normal and caesarian), parent’s education level (uneducated, primary education, secondary education, and higher education), father’s occupation (service, business, and farmer), mother’s occupation (housewife and service), family income (≤ 20,000Tk and > 20,000Tk per month), girl’s body mass index (BMI) (underweight (≤ 5th percentiles of BMI), normal weight (5th to 85th percentiles of BMI), overweight (85th to 95th percentiles of BMI), and obese (≥ 95th percentiles of BMI). The quantitative variables were number of siblings, order of birth, girl’s weight (in kg) and height (in cm), mother’s weight (in kg), height (in cm), BMI (kg/m^2^), and their age at menarche. Most of the independent variables had been selected on the basis of the previous studies [10, 12, 18].

#### Data collection procedure

All information of selected girls was gathered from respective school authorities. Girl’s current age was calculated by the difference between survey and birth date (considering nearest integer age category) and classified into one of the age categories. Digital scales and a portable stadiometer were used to measure their weight and height respectively. Measurement of individuals was taken without shoes and wearing light clothes using the techniques of Martin and Saller [19]. Height and weight were measured to the nearest 1 cm and 0.1 kg, respectively, and body mass index was calculated using the formula, BMI = weight (kg)/height (m)^2^. Mothers’ height and weight were also measured by the same procedures as applied for their girls.

#### Statistical analysis

Frequency distribution was used to determine the prevalence of age at menarche of girls with age 10 to 12 years. Independent sample *t* test was utilized in this study to find the difference between menarcheal and non-menarcheal girls for quantitative variables. Both simple and multiple binary logistic regression models were used to determine the effect of categorical independent factors on age at menarche among school girls (age 10 to 12 years). Standard error (SE) was used to detect the multicollinearity problem among independent factors for multiple logistic regression model, if the magnitude of the SE lies between 0.001 and 0.5, it is judged that there is no evidence of multicollinearity [20]. Finally, the Nagelkerke R^2^ value and Hosmer and Lemeshow test were utilized in this study to find the goodness of fit of multiple models. All statistical significance was accepted at *p* < 0.05. Statistical analyses were performed by using the SPSS software (IBM version-22).

## Results

A total number of 386 school girls aged 10–12 years were considered as sample for this study, among them 43 (11.1%), 139 (36.0%), and 204 (52.8%) girls were 10, 11, and 12 years old respectively. The number of girls in the youngest group was smaller compared to the other two groups. This happened because most of the girls in the youngest group felt shy to talk about their menarcheal status, and they refused to answer.

### Prevalence of age at menarche among school girls aged 10–12 years

**Fig. 1:**
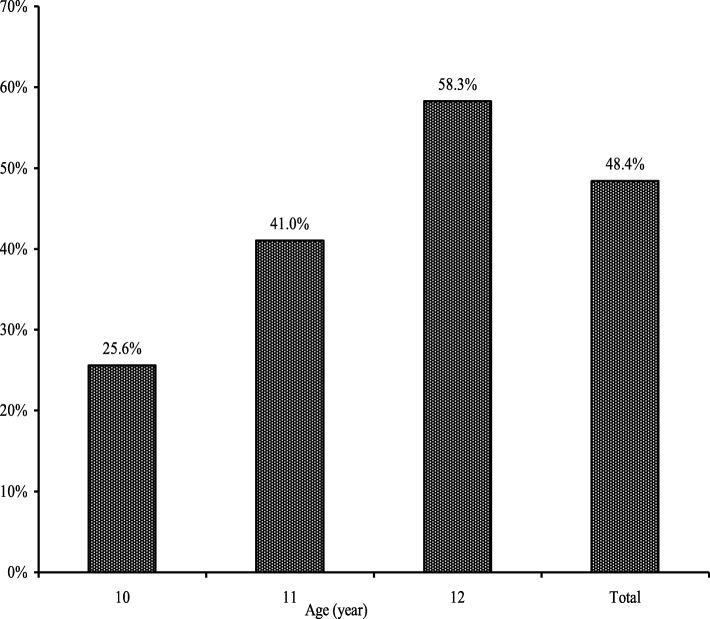
Prevalence of age at menarche among school girls in Rajshahi district

Among the selected girls, 187 (48.45%) already reached menarche, and 199 (51.55%) girls did not get menarche. The percentage of menarcheal girls was 25%, 41.0%, and 58.3% by their age 10, 11, and 12 respectively (Fig. 1).

### Proportions of menarcheal and non-menarcheal school girls by their parents socio-demographic factors and students’ body size

The proportion of menarcheal and non-menarcheal girls by their nutritional status and parents’ socio-economic and students’ BMI categories is presented in Table 1. The proportion of menarcheal girls in urban area (0.726) was significantly (*p* < 0.01) higher than rural (0.144). It was found that the proportion of menarcheal girls of higher educated parents was more than that of primary (*p* < 0.01) and secondary (*p* < 0.01) educated parents’ girls. The higher proportion of menarcheal girls was born by cesarean system than girls who were born by normal process (*p* < 0.01). The proportion of service holder fathers’ menarcheal girls was more than that of businessman (*p* < 0.01) and farmers’ (*p* < 0.01) girls. The proportion of menarcheal girls of service holder mothers was significantly (*p* < 0.01) higher than that of housewife mothers. It was observed that significantly (*p* < 0.01) more number of girls who lived in rich family reached menarche than that of comparatively poor family. It was noted that the proportion of menarcheal girls showed increasing tendency with increase in their body size, and obese girls reached menarche significantly higher than that of underweight (*p* < 0.01) and normal (*p* < 0.01) girls (Table 1).

**Table 1:** The proportions of menarcheal and non-menarcheal girls by their anthropometric and demographic variables

Variables	Did you attain menarche?					
	Yes			No		
	Proportion	95% CI		Proportion	95% CI	
		Lower	Upper		Lower	Upper
Type of residence						
Rural	0.144	0.093	0.208	0.856	0.792	0.902
Urban**	0.726	0.663	0.783	0.274	0.217	0.337
Mode of delivery						
Normal	0.438	0.373	0.504	0.562	0.496	0.627
Cesarean*	0.556	0.473	0.636	0.444	0.364	0.527
Father’s education level						
Uneducated	0.412	0.184	0.671	0.588	0.329	0.816
Primary	0.283	0.168	0.423	0.717	0.577	0.832
Secondary	0.286	0.179	0.413	0.714	0.587	0.821
Higher**	0.581	0.518	0.643	0.419	0.357	0.482
Mother’s education level						
Uneducated	0.357	0.128	0.649	0.643	0.351	0.872
Primary	0.302	0.183	0.443	0.698	0.557	0.817
Secondary	0.349	0.249	0.459	0.651	0.541	0.751
Higher**	0.584	0.518	0.648	0.416	0.352	0.482
Father’s occupation						
Service**	0.637	0.564	0.705	0.363	0.295	0.436
Business	0.385	0.301	0.474	0.615	0.526	0.699
Farmer	0.242	0.154	0.364	0.758	0.636	0.855
Mother’s occupation						
Housewife	0.438	0.383	0.495	0.562	0.505	0.617
Service**	0.696	0.573	0.801	0.304	0.199	0.427
Monthly family income						
≤ 20,000 (Taka)	0.340	0.273	0.413	0.660	0.587	0.727
>20,000 (Taka)**	0.621	0.550	0.689	0.379	0.311	0.450
Girls’ BMI category						
Underweight	0.286	0.173	0.422	0.714	0.578	0.827
Normal	0.414	0.350	0.479	0.586	0.521	0.650
Overweight	0.746	0.616	0.850	0.254	0.150	0.384
Obese**	0.853	0.689	0.950	0.147	0.050	0.311

*5% and **1% level of significantly higher

#### Mean difference in anthropometric and demographic variables between menarcheal and non-menarcheal girls

The mean weight of menarcheal girls (44.49 ± 9.61 kg) was significantly higher than that of non-menarcheal girls (35.46 ± 6.93 kg) (*p* < 0.01). Also, the mean height of menarcheal girls (146.14 ± 10.16 cm) was significantly (*p* < 0.01) higher than non-menarcheal girls (143.63 ± 7.96 cm). The number of siblings of non-menarcheal girls was significantly more than that of menarcheal girls (*p* < 0.01). It was found that the order of birth of non-menarcheal girls was significantly higher than that of menarcheal girls (*p* < 0.05). The mean weight and BMI of menarcheal girl’s mother were significantly higher than non-menarcheal girls’ mothers (*p* < 0.01), but the height of menarcheal girls’ mothers was lower than non-menarcheal girls’ mothers (*p* < 0.01). It was also found that mean age at menarche of non-menarcheal girl’s mother was significantly higher than that of menarcheal girl’s mother (*p* < 0.01) (Table 2).

**Table 2:** Mean difference in anthropometric and demographic variables between menarcheal and non-menarcheal girls

Variable	Did you attain menarche? (*N*)	Mean	SD	95% CI for Mean		*p* value
				Lower	Upper	
Girl’s weight (kg)	No (199)	35.46	6.93	34.49	36.43	0.001
	Yes (187)	44.49	9.61	43.11	45.88	
Girl’s height (cm)	No (199)	143.63	7.96	142.52	144.74	0.008
	Yes (187)	146.14	10.16	144.67	147.60	
Number of siblings	No (199)	2.43	0.91	2.30	2.55	0.003
	Yes (187)	2.16	0.82	2.04	2.28	
Order of birth	No (199)	1.87	0.96	1.74	2.00	0.013
	Yes (187)	1.65	0.79	1.53	1.76	
Mother’s weight (kg)	No (199)	57.40	8.91	55.85	58.96	0.001
	Yes (187)	62.51	8.23	61.33	63.70	
Mother’s height (cm)	No (199)	155.37	4.91	154.50	156.24	0.001
	Yes (187)	150.98	7.62	149.88	152.08	
Mother’s BMI (kg/m^2^)	No (199)	23.80	3.85	23.11	24.48	0.001
	Yes (187)	27.52	3.82	26.97	28.07	
Mother’s age at menarche (year)	No (199)	13.19	1.14	12.84	13.54	0.017
	Yes (187)	12.70	1.39	12.50	12.90	

#### Effects of parent’s socio-economic and girl’s nutritional status on their early age at menarche

The results of simple and multiple logistic regression models were interpreted by odds ratio (OR) and adjusted odds ratio (AOR) respectively. Simple logistic regression model demonstrated that obese school girls aged 10–12 years were more likely to reach menarche than that of girls having normal weight [OR = 0.122, 95% CI 0.0.045–0.325; *p* < 0.01] and underweight [OR = 0.069, 95% CI 0.023–0.210; *p* < 0.01]. It was noted that service holder mothers’ girls had 0.342 times higher chance to reach menarche than that of housewife mothers’ girls [OR = 0.342, 95% CI 0.195–0.597; *p* < 0.01]. Service holder [OR = 5.480, 95% CI 2.902–10.350; *p* < 0.01] and businessman [OR = 1.953, 95% CI 1.005–3.797; *p* < 0.01] fathers’ girls were more likely to attain menarche than that of farmers’ girls. Higher educated mothers’ girls were more likely to reach menarche 0.308 [OR = 0.308, 95% CI 0.162–0.586; *p* < 0.01] and 0.382 times [OR = 0.382, 95% CI 0.228–0.639; *p* < 0.01] higher than primary and secondary educated mothers’ girls respectively. Higher educated fathers’ girls had a more chance to get menarche than that of primary [OR = 0.285, 95% CI 0.149–0.544; *p* < 0.01] and secondary [OR = 0.288, 95% CI 0.158–0.526; *p* < 0.01] educated fathers’ girls. It was found that girls who were born by cesarean had 0.623 times higher chance to attain menarche than that of girls born by vaginal system [OR = 0.623, 95% CI 0.413–0.939; *p* < 0.01]. It was also observed that urban girls were more likely to get menarche than that of rural girls [OR = 0.063, 95% CI 0.037–0.108; *p* < 0.01] (Table 3). After controlling for potential confounders, multiple logistic regression model showed that obese school girls aged 10 to 12 years were 0.279 (0.075–0.986) and 0.248 (0.082–0.755) times higher (*p* < 0.05) chance to attain menarche than under and normal weight girls respectively. Also, urban school girls was more likely to get menarche than rural school girls [AOR = 0.012, 95% CI 0.003–0.047; *p* < 0.01]. Negelkerke R^2^ demonstrated that our selected model is able to explain the variation of outcome variable by 50.20%, and Hosmer and Lemeshow test showed that [Chi-square value = 3.005; *p* > 0.05] selected model was good fitted for the data (Table 3).

**Table 3:** Effect of parent’s socio-economic and girl’s nutritional status on age at menarche of school girls (age, 10–12 years)

Variables	OR (95% CI of OR)	AOR (95% CI of AOR)
Girls’ BMI category		
**Underweight**	0.069(0.023–0.210)**	0.279 (0.075–0.986)*
Normal weight	0.122(0.045–0.325)**	0.248 (0.082–0.755)*
Over weight	0.506(0.166–1.543)	0.594(0.169–2.085)
Obesity^**R**^		1.00
Mother’s occupation		
Housewife	0.342(0.195–0.597)**	0.753(0.320–1.773)
Survice^**R**^		1.00
Father’s occupation		
Service	5.480(2.902–10.350)**	1.396(0.402–4.843)
Business	1.953(1.005–3.797)**	1.281(0.457–3.595)
Farmer^**R**^		
Monthly family income		
≤ 20,000 (Taka)	0.315(0.208–0.477)**	1.499(0.893–2.680)
> 20,000 (Taka)^**R**^		
Mother’s education level		
Uneducated	0.396(0.129–1.219)	0.872(0.116–6.569)
Primary	0.308(0.162–0.586)**	0.829(0.232–2.964)
Secondary	0.382(0.228–0.639)**	0.889(0.360–2.197)
Higher^**R**^		
Father’s education level		
Uneducated	0.505(0.186–1.369)	4.698(1.844–7.200)
Primary	0.285(0.149–0.544)**	3.125(1.632–5.451)
Secondary	0.288(0.158–0.526)**	2.030(0.560–7.362)
Higher^**R**^		
Mode of birth		
Normal	0.623(0.413–0.939)*	1.142(0.645–2.023)
Cesarean^**R**^		
Type of Residence		
Rural	0.063(0.037–0.108)**	0.012(0.003–0.047)**
Urban^**R**^		
Model summary		
Negelkerke R^2^-value		0.502
Goodness of fit	Hosmer and Lemeshow test	Chi-square value = 3.005^n^

**1% and *5% level of significance

*R* reference case, *CI* confidence interval, *OR* odds ratio, *AOR* adjusted odds ratio

## Discussion

### Prevalence of age at menarche

Adolescent school girls (age, 10–12 years) were considered in this study to investigate their menarcheal status and factor influencing early age at menarche in Rajshahi District, Bangladesh. It was found that around 50% adolescent school girls aged 10–12 years reached menarche, among them 58.3% and 41.0% girls got menarche at age 12 and 11 years respectively, while only 25.6% school girls experienced menarche at their very early age (10 years). However, the frequency of the girls at age 10 years was smaller compared to the other groups. Due to lack of knowledge on menstruation or due to shyness and embarrassment, the frequency of early age at (10 year) menarche was very low in this study.

In other Bangladeshi studies, it was found that 35.7% urban school students got menarche at the age of 12 years [21]. The prevalence of menarcheal girls among Bangladeshi rural adolescent aged 10–17 was 37.8% [22]. In Pakistan, it was found that 0.8% girls attainted menarche at age 8 years; however, 46% girls got menarche in 10–14 year age group [23]. There are few studies available with age at menarche of adolescent girls in Bangladesh [21] and Bangladeshi female adults [15–22]. Study on prevalence of menarche and identify factors which were related to early onset of menarche among Bangladeshi adolescent school girls aged 10–12 years to compare menarcheal girls with non-menarcheal girls was poorly documented. Hossain et al. reported that the mean and median age at menarche of Bangladeshi university students (Birth-year cohorts from 1979 to 1986) were 13.12 ± 1.16 years and 13.17 years respectively [22]. Meng et al. reported that the age at menarche has declined worldwide [24]. Other studies reported that the average age of age at menarche among Pakistani girls was 11.73 years [20], Indian girls was 12.4 years [25], and the mean age at menarche of Nigerian school girls was 12 years [26].

### Menarcheal age and anthropometric measures

This study observed that taller and obese girls reached menarche in their early life than underweight or normal weight girls because body size parameters, such as weight or BMI and height, are strongly correlated with the age at menarche. Frisch and Revelle proposed a critical body weight and weight gain for the onset of menarche [27, 28]. Higher subcutaneous fat levels and BMI at prepubertal ages (5–9 years) are associated with increased likelihood of early (< 11 years) menarche [29]. Same result had been found in a South Korean study; Kim et al. found that the menarcheal girls aged 10–14 were taller and heavier than non-menarcheal girls among South Korean adolescent girls [30]. Also, they found that menarcheal girls had more BMI than non-menarcheal ones. Our results were also supported by an African study; Engidaw and Gebremariam found that Somalian adolescent refugee girls living in eastern Somali refugee camps who did not start menstruating were less likely to be thin/wasted compared to those who did [31]. For Okasha et al. who found that in female students at the University of Glasgow, age at menarche was positively associated with adult height and negatively associated with weight [32] and BMI; in Bangladeshi studies, Hossain et al. reported that age at menarche of Bangladeshi female students was negatively associated with adult BMI but positively associated with adult height [15]. These results were also supported by the findings of Chowdhury et al. who found that the age at menarche of Bangladeshi females was negatively associated with BMI and positively with height [22], and Ersoy et al. who reported an inverse relationship between age at menarche and post-menarcheal weight and BMI of Turkish female students [33].

### Menarcheal age and family socio-demographic factors

Logistic regression model demonstrated that school girls had less number of siblings experienced menarche earlier than their counterparts. Szwed et al. demonstrated that the girls from large families (four and more children) were the latest to cross the pubertal threshold on average at the age of 13.54 years [18]. It was observed that school girls aged 10–12 years who were living in urban areas and girls in urban living with rich family got menarche earlier compare to rural girls and they eat more nutritious food than rural girls. Consequently, they become more fatty; as a result, they got menarche earlier than rural girls. These results are in agreement with others finding [34]. The present study demonstrated that parents’ educational level and occupation have a significant influence on their daughter’s age at menarche. Also, we found that mothers’ BMI was an important factor of their daughter’s early menarche. Evidence for hereditary influences on the age at menarche comes from studies that show a trend for maternal age at menarche to predict daughter’s age at menarche [35]. In fact, approximately half of the phenotypic variation among girls from developed countries in the timing of menarche is due to genetic factors [4].

To the author’s knowledge, there have been no comparable studies on this in Bangladesh to date. However, in a similar study conducted on female university students in Portugal, Padez found no association between a girl’s age at menarche and her parent’s educational level and occupation [36]. It seems that parental education and employment does not have any direct effect on menarcheal age and might exert its effect indirectly through influencing family lifestyle [37]. The present study found that females from rural locations had a later age at menarche than those who spent their adolescence in urban areas. Identical results have been found in Portugal [36] and Spain [38]. It may be difficult to make conclusions based on multiple studies from different parts of the world and perhaps more local, regional studies should be conducted. This study only investigated the age at menarche and it is associated with some factors of the adolescent school girls. Other important factors that may be directly implicated in menstrual problems are birth weight [39], childhood living conditions [40], food habits in childhood [34], physical activity, lifestyle factors and nutrition [41], history of smoking, dietary habits, psychosocial stress, and dieting [42, 43].

## Conclusion

In this study, we investigated the menarcheal status among school girls aged 10–12 years in Rajshahi District, Bangladesh. The prevalence of age at menarche among school girls was 48.4%, and a remarkable number of girls (25.6%) got menarche at the age of 10. It was observed that taller and heavier girls got menarche earlier than their counterparts. The mean height and BMI of menarcheal girls’ mother were significantly higher than non-menarcheal girls’ mothers. Also, it was noted that comparatively heavier mothers’ girls got menarche earlier than their counterpart. High maternal BMI is a risk factor for daughter’s early onset of menarche. There was a positive association between mother’s age at menarche and their daughters’ age at menarche. Overnourished and urban girls were more likely to get menarche than undernourished and rural girls. Age at menarche of school girls was influenced by their parents’ socio-demographic and their anthropometric factors. Parent’s educational level and occupation have a significant influence on their daughter’s age at menarche. It was noted that, school girls had less number of siblings who experienced menarche earlier than their counterparts.

## Data Availability

The study was based on the primary data. The data will be provided when necessary.

## Abbreviations

BMI: Body mass index
SE: Standard error
OR: Odds ratio
AOR: Adjusted odds ratio
CI: Confidence interval
P: *P* value
